# The structure and morphologic changes of antennae of *Cyrtorhinus lividipennis* (Hemiptera: Miridae: Orthotylinae) in different instars

**DOI:** 10.1371/journal.pone.0207551

**Published:** 2018-11-26

**Authors:** Han-Ying Yang, Li-Xia Zheng, Zhen-Fei Zhang, Yang Zhang, Wei-Jian Wu

**Affiliations:** 1 Laboratory of Insect Ecology, South China Agricultural University, Guangzhou, China; 2 College of Agronomy, Jiangxi Agricultural University, Nanchang, China; 3 Plant Protection Institute, Guangdong Agricultural Science Academy, Guangzhou, China; Nanjing Agricultural University, CHINA

## Abstract

*Cyrtorhinus lividipennis* Reuter (Hemiptera: Miridae: Orthotylinae), including nymphs and adults, are one of the dominant predators and have a significant role in the biological control of leafhoppers and planthoppers in irrigated rice. In this study, we investigated the antennal morphology, structure and sensilla distribution of *C*. *lividipennis* in different instars using scanning electron microscopy. The antennae of both five different nymphal stages and adults were filiform in shape, which consisted of the scape, pedicel and flagellum with two flagellomeres. There were significant differences found in the types of antennal sensilla between nymphs and adults. The multiporous placodea sensilla (MPLA), basiconica sensilla II (BAS II), and sensory pits (SP) only occurred on the antennae of adult *C*. *lividipennis* of both sexes. Moreover, there was chaetica sensilla III (CHA III) only observed in males. Sixteen types of antennal sensilla were recorded altogether. They were microtrichia sensilla (MIC), three types of trichoidea sensilla (TRI I-III), three types of chaetica sensilla (CHA I-III), three types of basiconica sensilla (BAS I-III), two types of coeloconica sensilla (COE I and COE II), placodea sensilla (PLA), campaniform sensilla (CAM), MPLA, and SP. In the five different nymphal stages of *C*. *lividipennis*, the length of their antennae was significantly increased with the increase of the instar, as well as the number of the TRI II and TRI III. Moreover, sexual dimorphism usually occurred not only in the distribution (CHA III and SP) and the number of antennal sensilla (MIC, BAS II, TRI II, TRI III and MPLA), but also in the length of flagellum (F1 and F2). The possible functions of antennal sensilla are discussed. Those observations could contribute to a better understanding of the development of the olfactory system, and facilitate future studies on the antennal functions in *C*. *lividipennis*.

## Introduction

More than 3.5 billion people depend on rice (*Oryza sativa* L.) as their food staple, and more than 90% of the world’s rice is produced and consumed in Asia [1,2]. The rice planthoppers (Hemiptera: Delphacidae) including the brown planthopper (*Nilaparvata lugens* (Stål), white-backed planthopper (*Sogatella furcifera* (Horváth)), and small brown planthopper (*Laodelphax striatellus* (Fallén)), and the rice green leafhopper (*Nephotettix virescens* Distant (Homoptera: Euscelidae)), have become serious pests in most rice-producing countries in Asia [3–5]. Annual yield loss to rice caused by these pests, especially by planthoppers alone was one million tonne during 1970–1990 [6].

The mirid bug, *Cyrtorhinus lividipennis* Reuter (Hemiptera: Miridae: Orthotylinae), is widely distributed in rice fields and is an important predator that feeds on the eggs and young nymphs of rice planthoppers (*N*. *lugens*, *S*. *furcifera* and *L*. *striatellus*) and rice green leafhopper (*N*. *virescens*) [7–10]. In irrigated rice, *C*. *lividipennis* is one of the dominant predators and has a significant role in the biological control of leafhoppers and planthoppers [11,12]. Reyes and Gabriel [13] have reported that individual *C*. *lividipennis* nymphs and adults could suck an average of 7.5 eggs or 1.4 hoppers per day for a period of 14 days, and 10.2 eggs or 4.7 nymphs or 2.5 adults per day for a period of 10 days, respectively. Previous studies have confirmed that *C*. *lividipennis* can keep hopper populations at a low level [14].

Insect antennae are important sensory organs involved in habitat searching, host location, host discrimination, mating and oviposition [15–17], interspecific and intraspecific marking discrimination [18]. They carry a wide range of sensilla with different sensory modalities and can be categorized as chemo-, mechano-, thermo-, or hygro-receptors [19]. The olfactory system of natural enemies must accomplish several tasks [20]. Responses to volatile phytochemicals may be especially important in guiding enemies to their host or prey habitats [21,22]. Previous studies have demonstrated that *C*. *lividipennis* relies largely on herbivore-induced plant volatiles to identify eggs embedded in the rice stem tissues, and on pheromones to seek out mates [8,23,24]. To better understand the host location mechanism in *C*. *lividipennis*, we investigated the morphology, structure and sensilla distribution of *C*. *lividipennis* in different instars using scanning electron microscopy.

## Materials and methods

### Insects

*Cyrtorhinus lividipennis* was collected from rice field at the Experimental Farm of South China Agriculture University at Guangzhou, China (N123°10'7'', E113°21'27'') in July, 2017. The classification feature for *C*. *lividipennis* was listed in S1 File. The culture was fed on the rice seeding with brown planthopper eggs under the following conditions: 28 ± 1°C, 70 ± 5%, and 10:14 L:D.

### Scanning electron microscopy (SEM)

Antennae of newly hatched adult *C*. *lividipennis* and each instar of nymphs were excised under 80 × magnification (Carl Zeiss Microlmaging GmbH 37081 Göttingen, Germany) and fixed in 2.5% glutaraldehyde at 4°C for 24 h. The antennae were rinsed with 0.1 M phosphate buffer (PBS) for three times (40 min/time), then post-fixed in 1% osmium tetrachloride for 2.5 h. Subsequently, the specimens were rinsed with 0.1 M PBS for three times (10 min/time) again, and then dehydrated in a graded alcohol series from 30% (once for 10 min at 4°C), 50% (once for 10 min at 4°C), 75% (once for 10 min at 4°C), 80% (once for 10 min), 90% (once for 10 min), 100% (five times for 10 min each), and dried in a Bal-Tec CPD 030 critical point dryer 2 h. The treated specimens were anchored on the platform by double-sided adhesive tape in dorsal, ventral, and profile orientations. Finally, the specimens were coated with gold, and observed at 10 kV using a SEM (XL30, FEI, Holland and Nova Nano 430, FEI, Holland).

### Statistical analysis

The mean number of each sensilla type as directly calculated based on dorsal, ventral and two side profiles of antennae photomicrographs except microtrichia sensilla. The density of microtrichia sensilla was calculated from the numbers of sensilla in 10,000 μm^2^ from ten antennae of *C*. *lividipennis* of each stage (four locations were picked to count the density in 100 × 100 μm^2^ observation squares per antenna). Measurements were obtained from photomicrographs of at least ten individuals of the same type and a slide caliper (GB/T1214.1–1214.4) was used to calculate the means. Means were analyzed by general linear model (GLM) procedure and Tukey’s mean separation test. The mean number of sensory pits and multiporous plate sensilla between sexes was compared using nonparametric Mann-Whitney *U* test. Data were analyzed with SPSS 11.0 (http://www.spss.com).

### Terminology

In classifying sensilla, all of the terminology used in this work followed the methodology described by Zacharuk [15], Schneider [25], Dweck and Gadallah [26], Zhang et al. [27–29].

## Results

### General morphology of antennae

**Fig 1:**
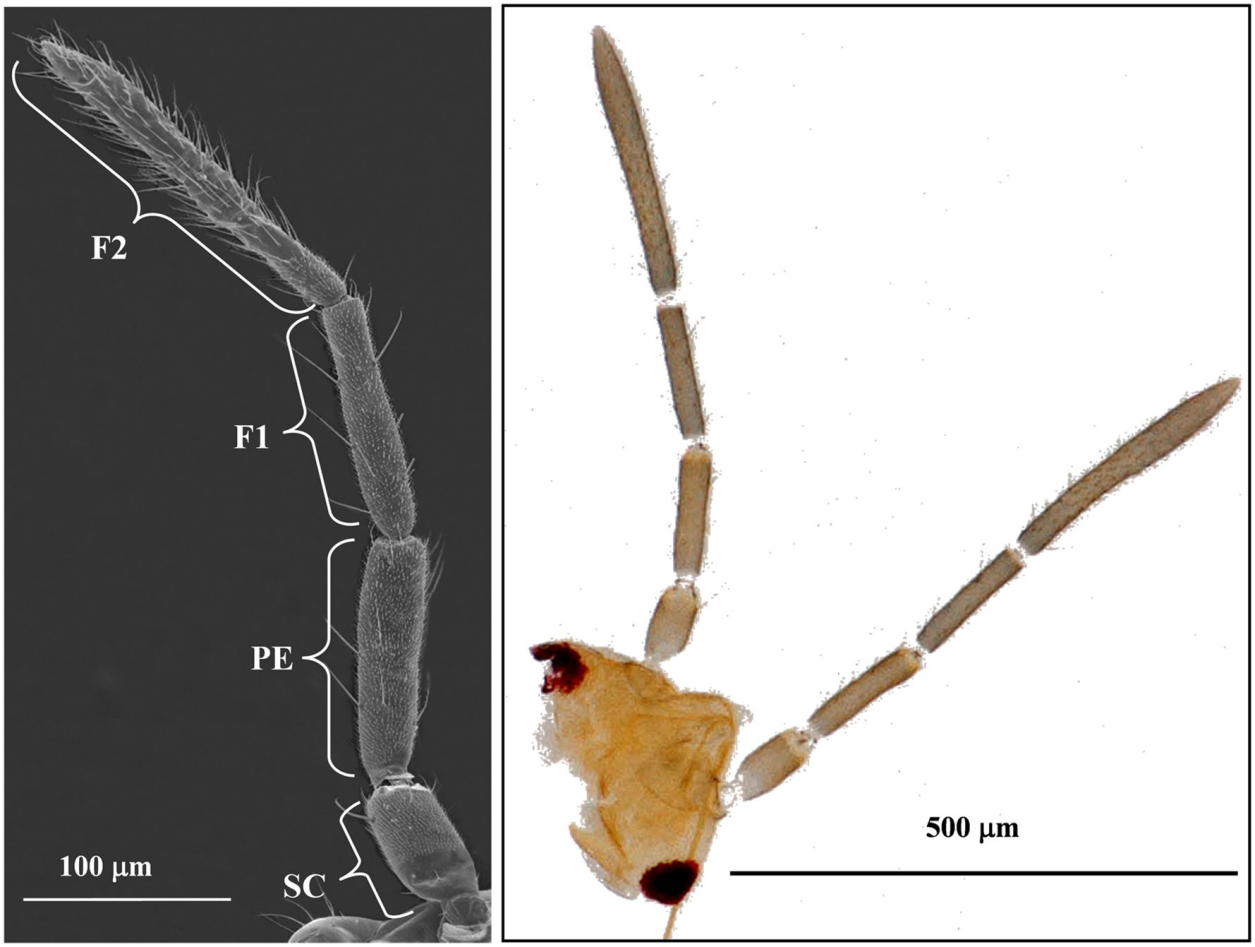
Antennae in first-instar nymph *Cyrtorhinus lividipennis*, showing scape (Sc), pedicel (Pe), and flagellum with two sub-segments (F1 and F2). The antennae of other four nymphal stages and adults were the same, only first-instar nymphal antennae are shown here.

The antennae of both five different nymphal stages and adults were filiform in shape and composed of three segments: the scape (Sc), the pedicel (Pe), and the flagellum composed in turn of two sub-segments (F1 and F2) (Fig 1). There were significant differences found in the length of *C*. *lividipennis* antennae of various stages (Table 1). The adult female antennae were the longest, but no difference with the fifth-instar nymphal and adult male antennae. In addition, the lengths of Sc and Pe in females were 257.68 ± 11.66 μm and 814.38 ± 19.49 μm, respectively, which were shorter than the males. However, the lengths of F1 and F2 in females were significantly longer than the males. There were significant differences observed in the length of *C*. *lividipennis* antennae of each nymphal stage. The length of the nymphal antennae was significantly increased with the increase of the nymphal instar. Similarly, the length of Sc, Pe, F1 and F2 were also increased with the increase of the nymphal instar. The length of each segment of the fifth-instar nymph was significantly longer than those of the other nymphal stages.

**Table 1:** Length (μm) (mean *±* SE, *n* = 10) of *C*. *lividipennis* antennae of various stages.

Instar	Sc	Pe	F1	F2	Total
1^st^	70.67±2.68d	123.28±2.23e	128.16±2.21d	239.18±4.83c	561.28±7.30e
2^nd^	108.63±4.65c	216.04±13.08d	201.96±11.04d	288.50±10.20c	815.12±30.15d
3^rd^	132.48±4.93c	273.23±7.12d	334.27±14.01c	371.66±5.13b	1111.63±21.23c
4^th^	181.54±6.49b	386.78±12.49c	457.02±17.83b	426.36±6.71b	1451.70±38.35b
5^th^	248.04±13.94a	690.94±37.49b	655.56±37.24a	519.33±15.33a	2060.30±87.05a
Adult female	257.68±11.66a	814.38±19.49a	665.71±41.02a	508.17±14.70a	2245.94±68.95a
Adult male	262.68±7.00a	879.56±12.61a	489.10±12.77b	426.49±26.02b	2057.84±44.76a

Means with same letters in the same column are not significantly different (GLM, Tukey, *P*>0.05).

### Sensillum type

**Fig 2:**
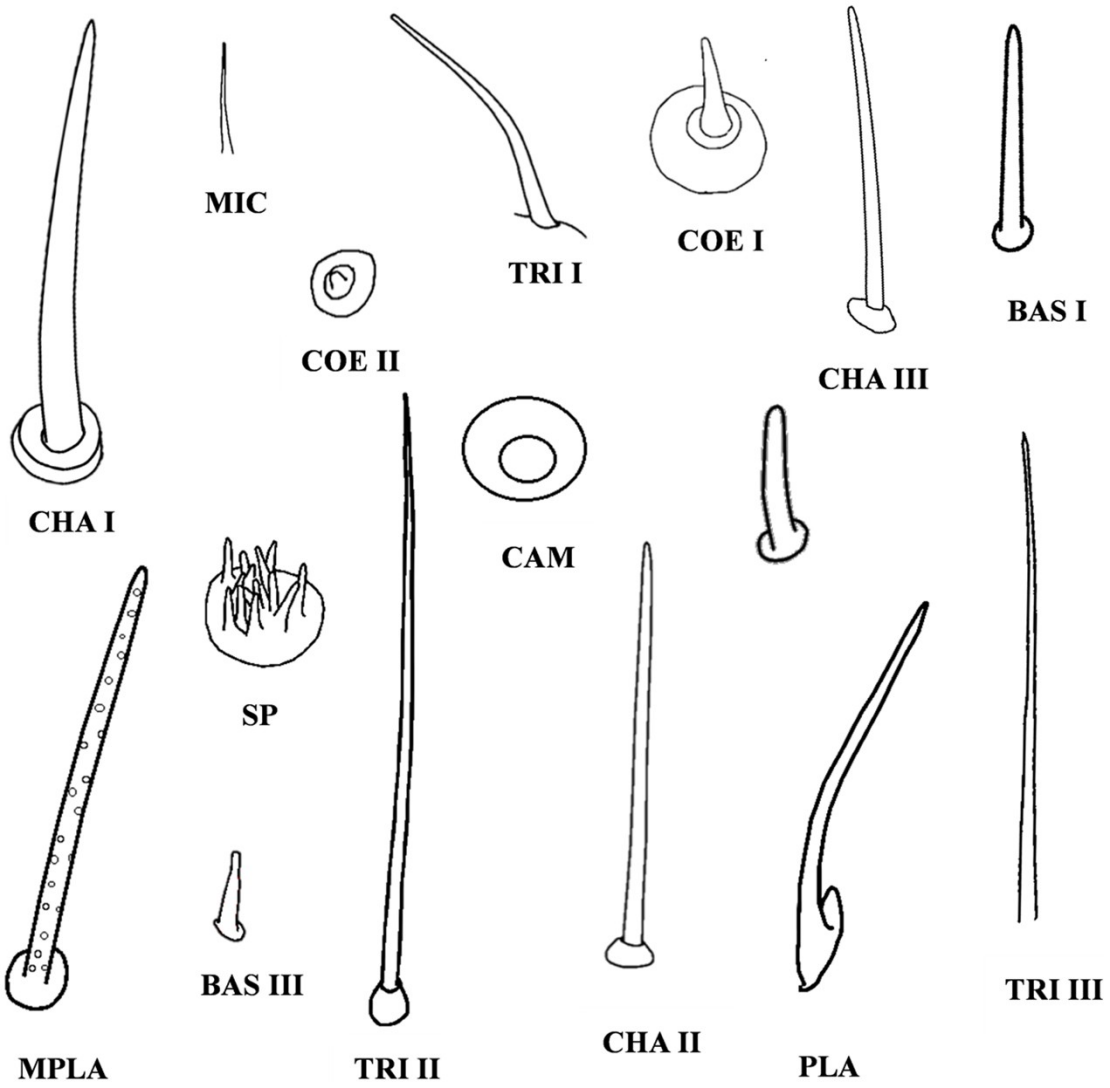
Diagrams of different types of sensilla on the antennae of *Cyrtorhinus lividipennis*. TRI I, TRI II and TRI III are trichoidea sensilla I, II and III; BAS I, BAS II and BAS III are basiconica sensilla I, II and III; MIC, microtrichia sensilla; CHA I, CHA II and CHA III are chaetica sensilla I, II and III; COE I and COE II are coeloconica sensilla I and II; PLA, placodea sensilla; MPLA, multiporous placodea sensilla; SP, sensory pits; CAM, campaniform sensilla.

**Fig 3:**
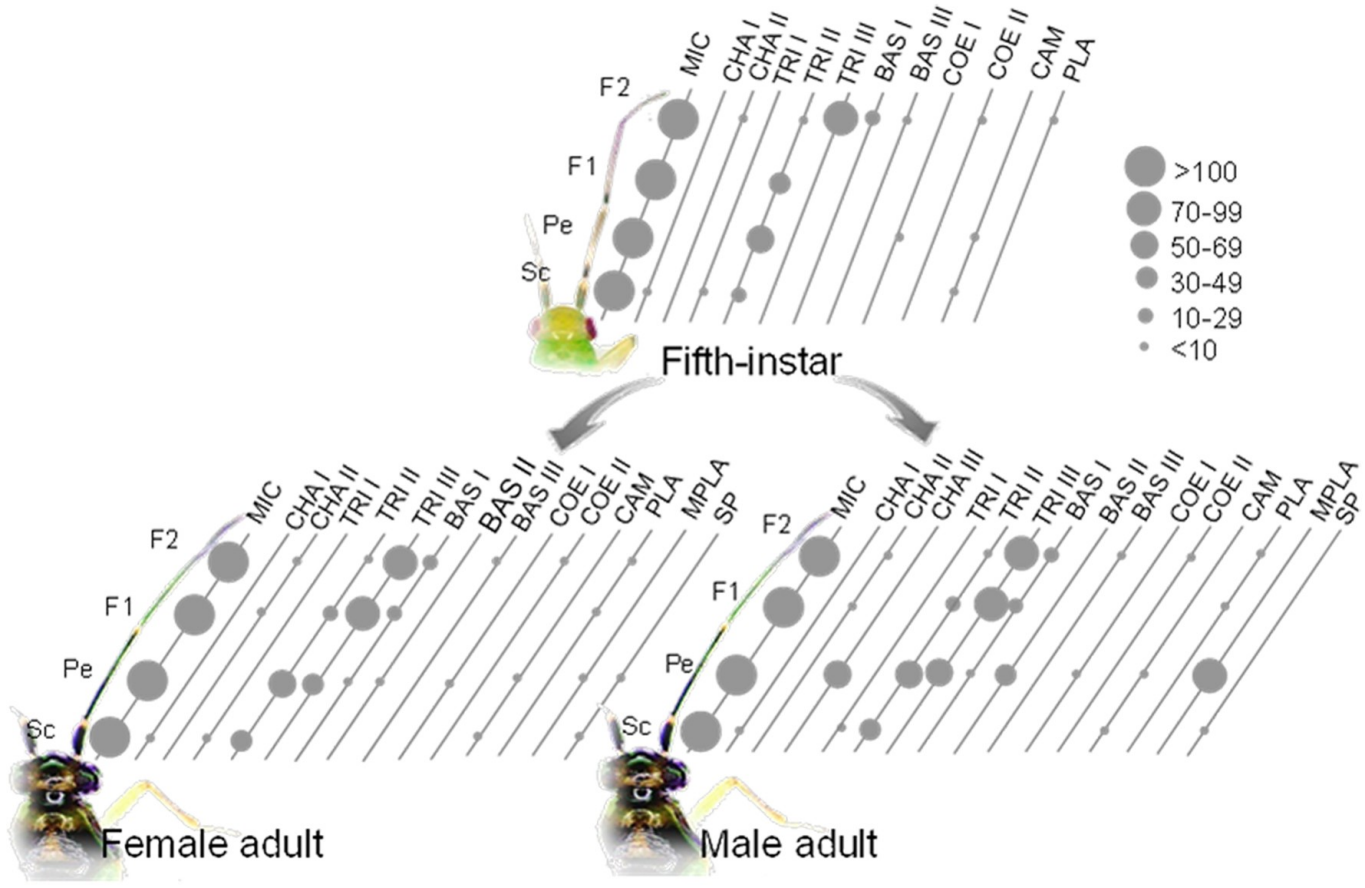
Sensilla distribution on the antennae in fifth-instar nymph, female and male adult of *C*. *lividipennis*. TRI I, TRI II and TRI III are trichoidea sensilla I, II and III; BAS I, BAS II and BAS III are basiconica sensilla I, II and III; MIC, microtrichia sensilla; CHA I, CHA II and CHA III are chaetica sensilla I, II and III; COE I and COE II are coeloconica sensilla I and II; PLA, placodea sensilla; MPLA, multiporous placodea sensilla; SP, sensory pits; CAM, campaniform sensilla. Sc, scape; Pe, pedicel; F1, first segment of flagellum; F2, second segment of flagellum. Scale indicates the number of sensilla.

Based on the morphological characteristics (Fig 2), the sensilla identified on the antennae of the five different nymphal stages of *C*. *lividipennis* could be classified into twelve types: microtrichia sensilla (MIC), three types of trichoidea sensilla (TRI I-III), two types of chaetica sensilla (CHA I and CHA II), two types of basiconica sensilla (BAS I and BAS III), two types of coeloconica sensilla (COE I and COE II), placodea sensilla (PLA), and campaniform sensilla (CAM). Especially, the multiporous placodea sensilla (MPLA), BAS II, and sensory pits (SP) only occurred on the antennae of adult *C*. *lividipennis* of both sexes. There were fifteen sensilla types identified on the female antennae: MIC, TRI I-III, CHA I and II, BAS I-III, COE I and II, PLA, MPLA, CAM, and SP. The sensilla which occurred on the female antennae were also found in males. Moreover, there was CHA III only observed in males (Fig 3).

### Morphology and structure of sensilla

#### Microtrichia sensilla

**Fig 4:**
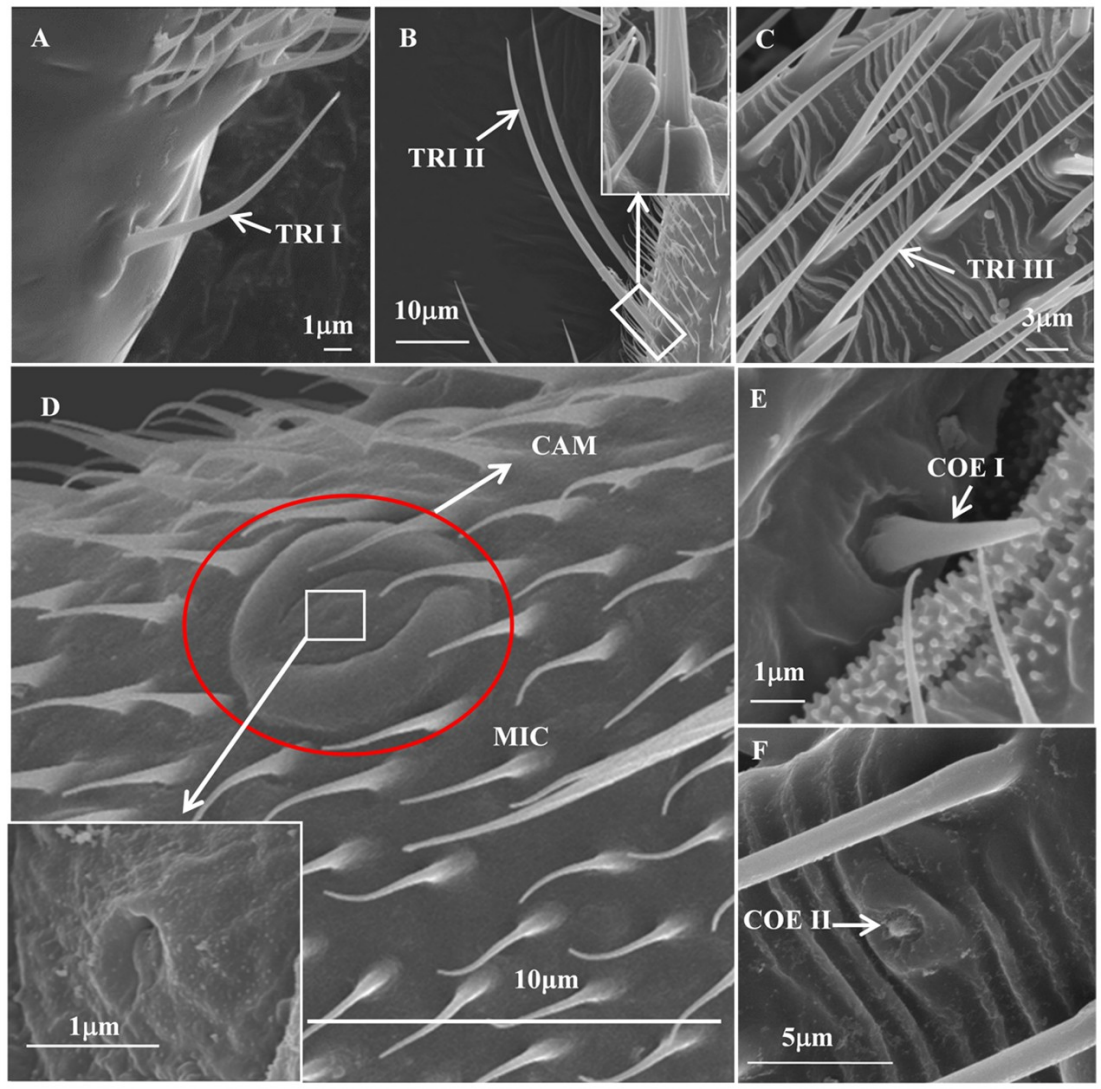
Scanning electron microscope (SEM) of sensilla on the antennae of first-instar nymph. (A) Trichoidea sensilla I on the scape. (B) Trichoidea sensilla II on the pedicel. Inset: The high magnification of the basal part of trichoidea sensilla II. (C) Trichoidea sensilla III on the second flagellum. (D) Microtrichia sensilla and campaniform sensilla on the scape. Inset: The high magnification of the central sunken of campaniform sensilla. (E) Coeloconica sensilla I on the pedicel. (F) Coeloconica sensilla II on the second flagellum. TRI I, II and III are trichoidea sensilla I, II and III; MIC, microtrichia sensilla; CAM, campaniform sensilla; COE I and II, coeloconica sensilla I and coeloconica sensilla II. These sensilla on *C*. *lividipennis* antennae at other stages were the same, only those in first-instar nymphs are shown here.

Microtrichia sensilla (MIC) were the most abundant and had a wide distribution range over the entire antennae of various stages. They were short and straight hairs with smooth surface, measuring 4.11 ± 0.24 μm in length with a basal diameter of 0.40 ± 0.02 μm. There was no socket at the basal part of each MIC, and the sharp tip was slightly curved toward the antennal shaft (Fig 4D). The density (per 10,000 μm^2^) of MIC on each segment of the antennae was significantly higher in first-instar nymphs than those in fifth-instar nymphs, females and males (Table 2). In addition, the density (per 10,000 μm^2^) of MIC on each segment of adult male antennae (except F2) was the fewest.

**Table 2:** Density (/10,000 μm^2^) (mean ± SE, *n* = 10) of microtrichia sensilla on *C*. *lividipennis* antennae of various stages.

Instar	Sc	Pe	F1	F2
1^st^	1627.00±108.77a	1399.30±148.16a	1013.00±17.52ab	805.60±60.98a
2^nd^	1248.10±50.46b	1133.40±131.56ab	992.50±132.25abc	693.40±37.42ab
3^rd^	1576.90±84.97a	1206.10±78.12ab	956.60±53.12abc	597.00±53.03bc
4^th^	1343.40±57.71b	1101.50±61.59b	832.40±57.87bc	573.00±35.70bc
5^th^	1003.80±91.58c	1168.70±72.15ab	1091.40±58.46a	410.20±20.35c
Adult female	1176.00±58.29bc	753.40±46.43c	795.70±44.00c	549.30±24.78bc
Adult male	967.20±69.92c	262.90±26.31d	183.10±8.35d	591.00±47.72bc

Means with same letters in the same column are not significantly different (GLM, Tukey, *P*>0.05).

#### Trichoidea sensilla

Trichoidea sensilla (TRI) were divided into types I, II, and III based on different shapes. TRI I were scattered on the Sc of *C*. *lividipennis* antennae of each development stage. They were positioned in cuticular socket with smooth surface and gradually bent toward the apex of the segment, ended with a sharp tip (Fig 4A). One to three TRI I were found and measured 11.83 ± 0.52 μm in length, 0.90 ± 0.02 μm in width.

Trichoidea sensilla II (TRI II) were widely distributed over the entire antennae of each development stage. They were also inserted into the socket and exhibited a longitudinally grooved shaft, sat at an approximate 45-degree angle to the longitudinal axis of the antennae (Fig 4B, inset of Fig 4B). TRI II were longer and straighter than TRI I, 31.72 ± 1.39 μm in length and 1.56 ± 0.06 μm in width. They were mainly distributed on the Pe and F1, followed by the Sc, with a few on the F2 (Table 3). In general, the numbers of TRI II on each segment were significantly increased with the increase of the nymphal instar. The total number of TRI II on the female antennae was significantly more than that in the other stages.

**Table 3:** Number (mean ± SE, *n* = 10) of trichoidea sensilla II on *C*. *lividipennis* antennae of various stages.

Instar	Sc	Pe	F1	F2	Total
1^st^	0.80±0.25f	12.10±0.46e	13.40±0.56cd	3.80±0.25ab	31.10±0.75f
2^nd^	4.00±0.60e	13.10±0.72e	12.30±0.60d	3.20±0.29b	34.60±1.44f
3^rd^	6.40±0.43d	15.60±0.48e	16.60±1.13c	3.90±0.31ab	45.50±1.34e
4^th^	15.60±0.48c	33.80±1.31d	29.10±1.22b	3.60±0.27ab	86.10±2.08d
5^th^	22.10±0.82b	57.50±2.06c	34.40±1.69a	3.70±0.21ab	122.80±3.54b
Adult female	32.10±1.08a	67.90±2.76a	29.60±2.24b	3.70±0.15ab	139.30±3.74a
Adult male	30.10±1.34a	50.30±1.42b	12.20±0.68d	4.10±0.23a	103.70±2.32c

Means with same letters in the same column are not significantly different (GLM, Tukey, *P*>0.05).

Trichoidea sensilla III (TRI III) were found on the F2 of the nymphal antennae, the Pe, F1 and F2 of the adult antennae. Similar to TRI II, the TRI III were long, straight and tapering tip. However, they were nonsocketed and slightly flat with smooth surface (Fig 4C). TRI III had a mean length and width of 38.29 ± 1.57 μm and 1.21 ± 0.04 μm, respectively. There was no difference in the number of TRI III on the F1 and F2 of the adult antennae, but a significant difference on the Pe (Table 4). The total number of TRI III was increased with the increase of the instar, and the males had the most TRI III.

**Table 4:** Number (mean ± SE, *n* = 10) of trichoidea sensilla III on *C*. *lividipennis* antennae of various stages.

Instar	Pe	F1	F2	Total
1^st^	—	—	70.80±1.93c	70.80±1.93d
2^nd^	—	—	71.80±1.73c	71.80±1.73d
3^rd^	—	—	90.50±1.71b	90.50±1.71c
4^th^	—	—	99.00±3.11a	99.00±3.11c
5^th^	—	—	99.40±2.29a	99.40±2.29c
Adult female	32.30±1.72b	94.60±2.88a	97.90±2.18a	224.80±3.89b
Adult male	64.00±2.91a	97.50±2.66a	95.70±2.19ab	257.20±4.96a

Means with same letters in the same column are not significantly different (GLM, Tukey, *P*>0.05). “–” indicates absent.

#### Chaetica sensilla

**Fig 5:**
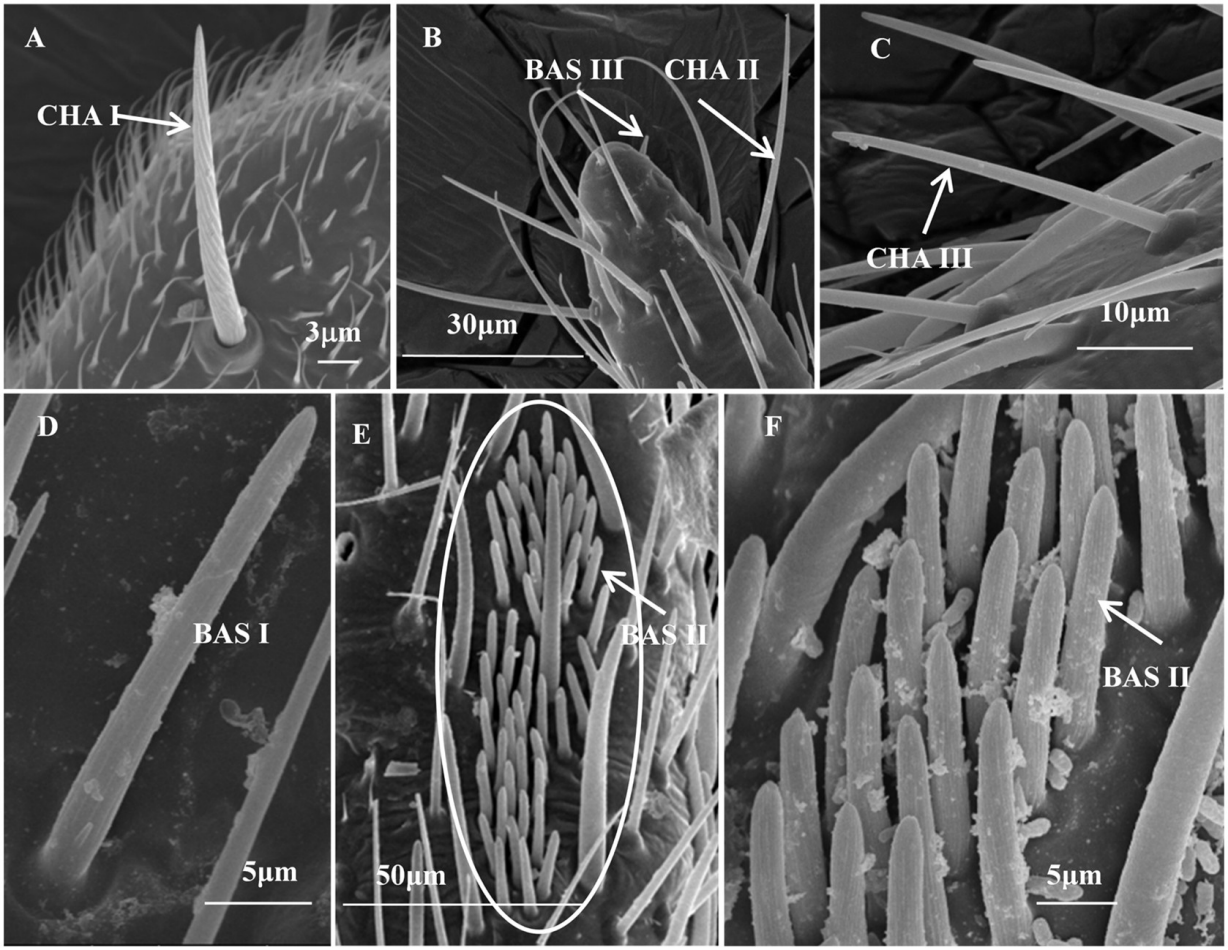
Scanning electron microscope (SEM) of sensilla on the adult male *C*. *lividipennis* antennae. (A) Chaetica sensilla I on the scape. (B) Chaetica sensilla II and basiconica sensilla III on the terminal antennae. (C) Chaetica sensilla III on the pedicel. (D) Basiconica sensilla I on the second flagellum. (E&F) Basiconica sensilla II on the pedicel. CHA I, II and III are chaetica sensilla I, II and III; BAS I, II and III are basiconica sensilla I, II and III. These sensilla on *C*. *lividipennis* antennae at other stages were the same, only those in adult males are shown here.

Chaetica sensilla I (CHA I) occurred on the Sc of *C*. *lividipennis* antennae of each development stage. There were one to three CHA I on the middle part of the Sc of each antennomere. They were strong and straight, near-vertical to the longitudinal axis of the antennae and sharp-tipped with strong longitudinal grooves (Fig 5A). They were inserted into a flexible cuticular socket, and measured 40.34 ± 2.56 μm in length and 3.82 ± 0.15 μm in basal diameter.

Chaetica sensilla II (CHA II) were found on the F2 of the nymphal antennae, the F1 and F2 of the adult antennae. They also had a sharp-tipped with strong longitudinal grooves and inserted into a cuticular socket (Fig 5B). They formed an approximate 70-degree angle to the longitudinal axis of the antennae. There was no difference in the number of CHA II on the antennae of *C*. *lividipennis* of each development stage (Table 5). CHA II were much more in number compared to the CHA I and shorter in length (38.85 ± 0.94 μm) with a narrow basal diameter (1.70 ± 0.08 μm).

**Table 5:** Number (mean ± SE, *n* = 10) of chaetica sensilla II and III on *C*. *lividipennis* antennae of various stages.

Instar	CHA II		CHA III
	F1	F2	Pe
1^st^			
2^nd^	—	5.00±0.37a	—
3^rd^	—	5.30±0.34a	—
4^th^	—	5.30±0.58a	—
5^th^	—	4.80±0.44a	—
Adult female	—	5.70±0.37a	—
Adult male	5.20±0.66a	5.30±0.42a	—
	5.30±0.52a	6.10±0.31a	78.90±4.00

Means with same letters in the same column are not significantly different (GLM, Tukey, *P*>0.05). “–” indicates absent.

Chaetica sensilla III (CHA III) were only observed on the Pe near the flagellar base on adult male antennae. They were straight and positioned in cuticular socket with grooved surface (Fig 5C), measuring 30.10 ± 1.63 μm in length and 1.57 ± 0.05 μm in basal diameter. The number of CHA III was the most among the three types of CHA, ranged from 60 to 96.

#### Basiconica sensilla

Basiconica sensilla I (BAS I), similar to TRI III, were also found on the F2 of the nymphal antennae, the Pe, F1 and F2 of the adult antennae. No socket but a cuticle-depressed was noticed. They were short and small with a slightly blunt tip and grooved surface (Fig 5D). The adult females and males had significantly more BAS I than the nymphs, but no difference on the F2 of *C*. *lividipennis* antennae of various stages (Table 6). The sensilla measured 9.88 ± 0.21 μm in length and 1.54 ± 0.08 μm in basal diameter.

**Table 6:** Number (mean ± SE, *n* = 10) of basiconica sensilla I and II on *C*. *lividipennis* antennae of various stages.

Instar	BAS I				BAS II
	Pe	F1	F2	Total	Pe
1^st^	—	—	21.10±1.21a	21.10±1.21b	—
2^nd^	—	—	18.80±1.26a	18.80±1.26b	—
3^rd^	—	—	18.90±1.20a	18.90±1.20b	—
4^th^	—	—	20.30±0.83a	20.30±0.83b	—
5^th^	—	—	21.90±0.74a	21.90±0.74b	—
Adult female	8.10±0.55a	12.50±0.98a	20.50±0.79a	41.10±1.48a	5.10±0.38b
Adult male	6.80±0.61a	14.80±0.85a	19.70±1.16a	41.30±1.83a	36.60±1.58a

Means with same letters in the same column are not significantly different (GLM, Tukey, *P*>0.05). “–” indicates absent.

Basiconica sensilla II (BAS II) had an almost similar morphology with BAS I, relatively longer as compared to BAS I, and measured 10.22 ± 0.29 μm in length. Only one cluster of BAS II was concentrated on the “sensillar field” near the apical socket of the antennal Pe near the expanded F1 base in adult females and males (Fig 5E and 5F). There were at most seven BAS II per cluster in females, while the males had significantly more BAS II per cluster ranged from 30 to 44 (Table 6).

Basiconica sensilla III (BAS III) were distributed on the terminal antennae of *C*. *lividipennis* of various stages. They were thick and strong staff with blunt tip, smooth surface, and directly connected to the cuticle without cuticular socket (Fig 5B). Only two BAS III were found and measured 2.76 ± 0.10 μm in length and 1.09 ± 0.04 μm in basal diameter.

#### Coeloconica sensilla

Coeloconica sensilla I (COE I) had a basal round sensory structure, and the ambient cuticle was protuberant with an inner and outer diameter of 2.38 ± 0.98 μm and 5.18 ± 0.28 μm, respectively (Fig 4E). Notably, their central pegs were cone-shaped structure measured 8.03 ± 0.34 μm in length and 1.43 ± 0.07 μm in basal diameter. Two COE I were found on the basal part of Pe of *C*. *lividipennis* antennae of each development stage.

Only one coeloconica sensilla II (COE II) was present on the mid-dorsolateral surface of the last flagellar segment of *C*. *lividipennis* antennae of each development stage. Morphologically they were almost identical to the COE I, except the central pegs. The tiny central peg of COE II was protruded which was slightly higher than its ambient cuticle, and embedded in a pit (Fig 4F).

#### Placodea sensilla

**Fig 6:**
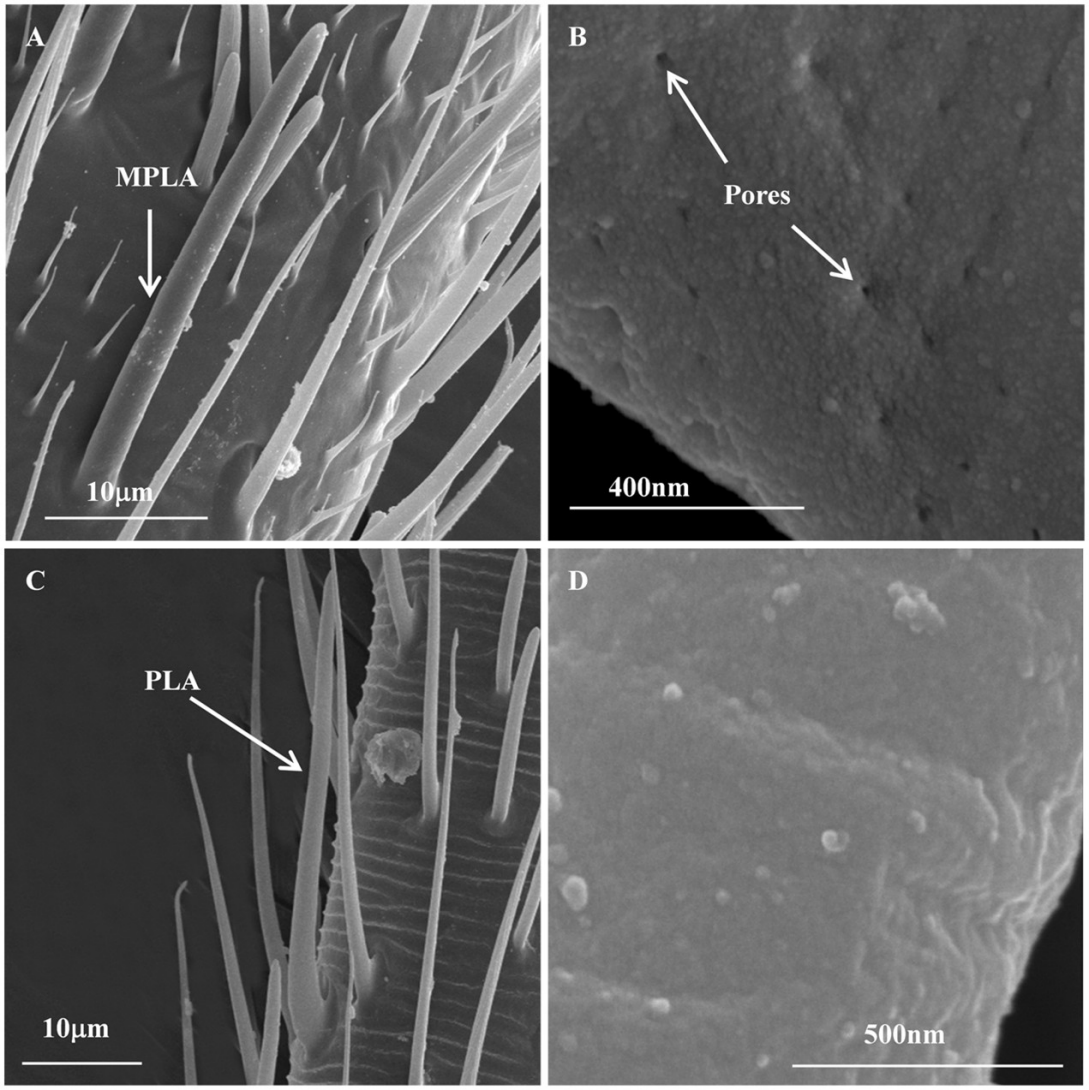
Multiporous placodea sensilla and placodea sensilla on the adult female *C*. *lividipennis* antennae. (A) Multiporous placodea sensilla on the pedicel. (B) The high magnification of MPLA, showing the pores (C) Placodea sensilla on the first flagellum. (D) The high magnification of PLA, showing the surface without pore. MPLA, multiporous placodea sensilla; PLA, placodea sensilla. MPLA and PLA in males or nymphs were the same, only those in females are shown here.

Placodea sensilla (PLA) were elongate, plate-like sensory organs distributed on the F2 of the nymphal antennae, the F1 and F2 of the adult antennae. Each sensillum arose from a cuticle-depressed structure (Fig 6C). The PLA exhibited a smooth surface with no pore (Fig 6D). They gradually tapered the apex and were generally aligned parallel with the antennal axis. The number of PLA in adults was significantly more than that in nymphs, but no difference between both sexes and nymphs (Table 7). The PLA had a mean length and width of 37.39 μm and 2.43 μm, respectively.

**Table 7:** Number (mean ± SE, *n* = 10) of placodea sensilla on *C*. *lividipennis* antennae of various stages.

Instar	F1	F2	Total
1^st^	—	0.90±0.28b	0.90±0.28b
2^nd^	—	2.20±0.25a	2.20±0.25b
3^rd^	—	1.70±0.26ab	1.70±0.26b
4^th^	—	0.80±0.36b	0.80±0.36b
5^th^	—	1.60±0.52ab	1.60±0.52b
Adult female	6.90±0.92a	2.30±0.45a	9.20±0.89a
Adult male	7.00±0.56a	2.80±0.55a	9.80±0.83a

Means with same letters in the same column are not significantly different (GLM, Tukey, *P*>0.05). “–” indicates absent.

#### Multiporous placodea sensilla

Multiporous placodea sensilla (MPLA) were only distributed on the Pe of the adult antennae. They had the similar morphology with PLA, but they were positioned close to the antennal surface and covered in numerous pores (Fig 6A and 6B). Notably, the number of MPLA in males was more than ten times than that in females (Table 8). However, the width of MPLA in females was significantly bigger than males. No difference was observed in the length between sexes.

**Table 8:** Numbers (mean ± SE, *n* = 10) of multiporous placodea sensilla on *C*. *lividipennis* antennae of both sexes.

Sexes	Numbers	Length (mm)	Width (mm)
Adult female	7.60±1.22b	35.98±1.10a	3.11±0.25a
Adult male	81.00±1.20a	37.48±0.61a	2.98±0.11b

Means with same letters in the same column are not significantly different (*P*>0.05) in Mann–Whitney *U* test.

#### Campaniform sensilla

Campaniform sensilla (CAM) were a dome-shaped sensory structure distributed on the Sc and Pe of *C*. *lividipennis* antennae of each development stage. The ambient cuticle was protuberant with smooth surface (Fig 4D). The CAM had a 7.42 ± 0.38 μm basal diameter, and the central conelet was oval with smooth surface and a tiny sunken, measuring 3.52 ± 0.16 μm in basal diameter. There was a pore at the central conelet of CAM (Insert of Fig 4D). Two or three CAM were found in nymphs, one on the basal part of Sc, one or zero on the basal part of Pe and one on the terminal part of Pe. Two or four CAM were found in females, one on the basal part of Sc, one or two on the central section of Sc and one on the terminal part of Pe. In males, two CAM were found, one on the basal part of Sc and one on the terminal part of Pe.

#### Sensory pits

**Fig 7:**
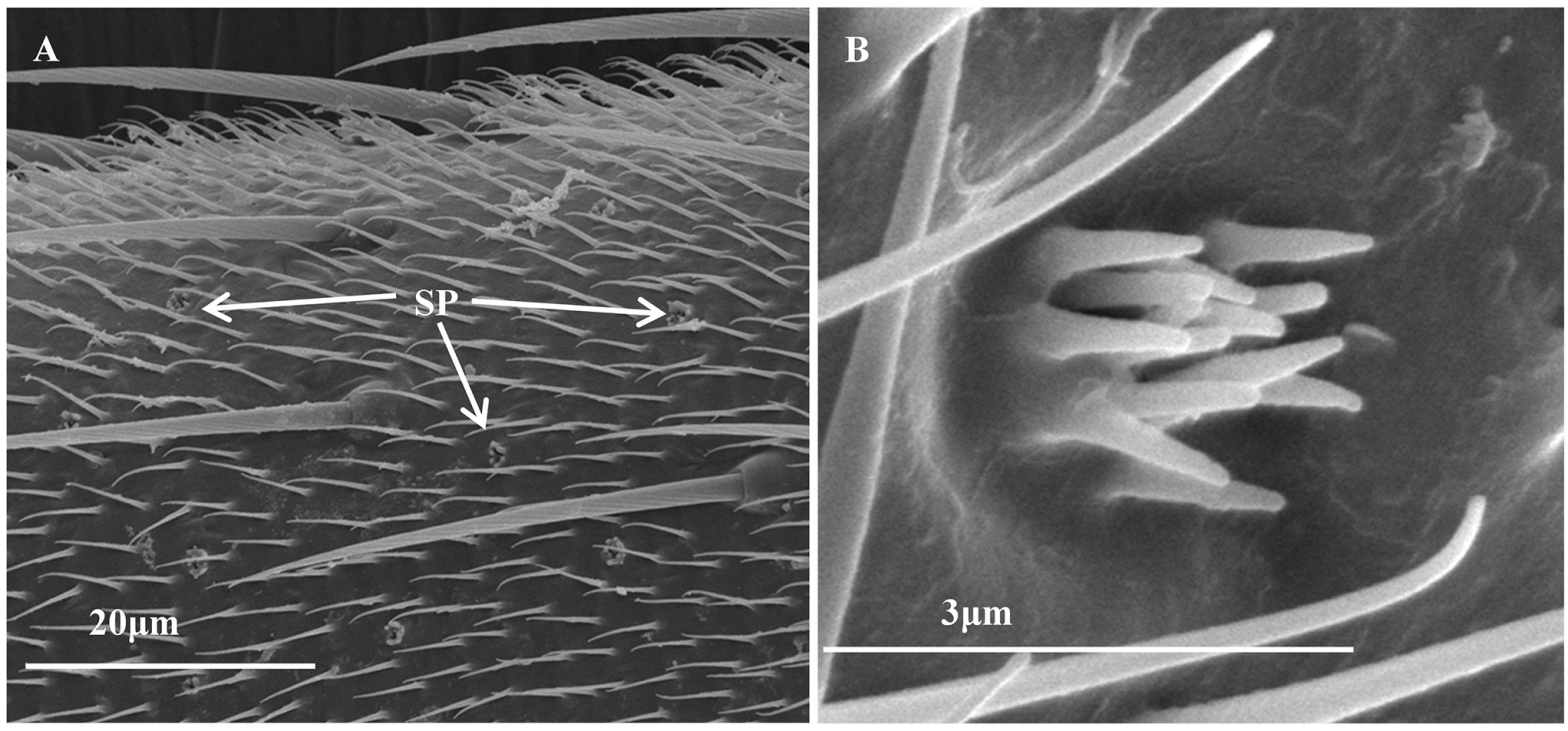
Sensory pits on the adult female *C*. *lividipennis* antennae. (A) Sensory pits on the scape. (B) The high magnification of sensory pits. SP, sensory pits. SP in males were the same, only those in females are shown here.

Sensory pits (SP) were usually circular sunken pits with microtriches structures (Fig 7A and 7B), on average diameter of 1.52 ± 0.05 μm. They were located mainly on the Sc and Pe in females, as well as on the Sc in males. Only one SP was found on the Pe of adult female antennae, and the number in SP exhibited no difference between sexes (Table 9).

**Table 9:** Numbers (mean ± SE, *n* = 10) of sensory pits on *C*. *lividipennis* antennae of both sexes.

Sex	Sc	Pe	Total
Female adult	8.60±0.85a	1.00±0.63	9.60±1.31a
Male adult	10.40±0.97a	—	10.40±0.97a

Means with same letters in the same column are not significantly different (*P*>0.05) in Mann–Whitney *U* test. “–” indicates absent.

## Discussion

In Heteroptera, antennal sensilla have been studied for many species belonging to different families [30–38]. Morphology and ultrastructure of the antennae and various antennal sensilla of *C*. *lividipennis* in different instars were studied. The antennae of *C*. *lividipennis* in different instars were all composed of an Sc, Pe and segmental flagellum. In general, most described types of antennal sensilla in Hemiptera as well as in other insects groups, are similar to the types of sensilla presented in this study. Sixteen types of antennal sensilla were observed altogether (Fig 3). In the five different nymphal stages of *C*. *lividipennis*, the length of their antennae was significantly increased with the increase of the instar, as well as the number of the TRI II and TRI III. Moreover, sexual dimorphism usually occurred not only in the distribution and number of antennal sensilla, but also in the length of the flagellum. The CHA III was only found in males, while the SP occurred on the Pe in females, not in males. In general, males had a larger number of the MPLA, BAS II and TRI III, but a smaller number of the TRI II and MIC on the Pe and F1 than in females (*P*<0.05). The average length of the F1 and F2 in females was significantly longer than that in males.

The MIC was the most widely distributed sensilla over the entire antennae of various stages. MIC described in this study was similar to those on the antennae of whiteflies [39] and *Habrobracon hebetor* [26], as well as on the maxillary palpi of several Diptera families [27,40,41]. The hair-like MIC is consistent with the comprehensive description of these sensilla by Zhang et al. [42], which they considered to have a mechanosensory function, may also be mechanoreceptors.

Three types of trichoidea sensilla were present on *C*. *lividipennis* antennae in different instars. The number of TRI II in females was significantly more than the males, while in the opposite case of TRI III. We found that TRI I (Fig 4A) on the antennal Sc were extremely similar to Böhm bristles, which are probably present in analogous locations in various insects [25,32,34,38]. Previous studies have demonstrated that this kind of sensilla is considered to be mechanoreceptors on the antennae, and presumably function as propriceptors which perceive antennal position [25,43]. TRI II with grooves on the surface (Fig 4B) and TRI III without any specialized raised socket (Fig 4C), were similar to other Hemiptera species, such as *Riptortus pedestris* [35] and *S*. *furcifera* [34]. According to the descriptions, TRI II and TRI III may be considered to be mechanoreceptors, chemoreceptors, or even as thermoreceptors or hygroreceptors [35,44].

The CHA I and II were found on the Sc and flagellum of *C*. *lividipennis* antennae of each development stage, respectively. They were the longest antennal sensilla in *C*. *lividipennis* as they would be the first sensilla to contact external objects, and may function as tactile mechanoreceptors [33,45–47]. CHA III occurred only on the Pe of adult male antennae, and was much more than CHA I and II in number (Fig 5C, Table 5). Several studies have reported that sexual dimorphism in antennae of insects is moderated and probably related to different functions and/or roles between sexes [48–51]. For male *C*. *lividipennis*, to success in finding and mating with the females must be one of the most important behaviors. Thus, based on the location and morphology, CHA III may integrate mechanosensory with chemosensory functions.

The BAS I and TRI III exhibited the same distribution on the antennae of *C*. *lividipennis*. BAS I and II were similar in appearance to those described for *Euschistus heros*, *Edessa meditabunda* and *Piezodorus guildinii* (Hemiptera: Pentatomidae) [31], and *R*. *pedestris* (Hemiptera: Alydidade) [52]. A larger numbers of BAS II in males may be also related to finding and mating with the females. Sensilla basiconica are structurally and functionally similar in most insect species studied [25]. Previous studies suggested that the BAS I with non-porous cuticular of the grooved surface should be a gustative function [53,54]. Very small and rare BAS III on the apical flagellomere in *C*. *lividipennis* resembled the “s.b.4”, “sensilla basiconica 4” and “sensilla basiconica types 3” in the same area of the antennae of the ground beetles *Bembidion properans* [55], *B*. *lampros* [56] and *Platynus* dorsalis [57], respectively. However, its function is not known.

COE were found in many Hemiptera across several families, such as Pentatomidae [31,58], Pyrrochoridae [59], Dinidoridae [52], Tropiduchidae [32], Coreidae [60], Aleyrodidae [33,61]. According to Ruchty et al. [62], COE serve as chemoreceptors that respond to air temperature changes in social insects. Pophf [63] has reported that COE with receptor neurons may respond to host plant volatile compounds in some lepidopterans. In homopterans, they function as hygroreceptors preventing desiccation of the antennae [64]. In this study, the COE I and II were found in very low numbers and any of the aforementioned functions can be presumed.

In the mirid bug *C*. *lividipennis*, male and female antennae were equipped with significantly more PLA than the nymphal antennae (Table 7). The PLA were similar in appearance to those described for *Aleurodicus dispersus* [33]. Their specific functions were yet to be confirmed electrophysiologically because of lacking a multiple cuticular system. Compared to PLA, the MPLA with numerous pores were relatively common in parasitic Hymenoptera, which may play a role in host location to detect the host-related semiochemicals [45,53,65,66]. In this study, the MPLA were only found in adult females and males. Moreover, the number of MPLA in males was significantly more than that in females (Table 8), which may suggest potential functions of these types in chemical communication during its precopulatory and copulatory activities [67,68]. In the family Miridae (mirid bugs), the sex pheromones are usually secreted by the females, to attract the males for mating [69–74].

CAM have been reported in all parts of the body regions of insects, including halters, legs, bases of wings, mouthparts, and even eyes [25,32,74]. They were found in many Hemiptera insects, including four Australian spittlebug species [75], *Zema gressitti* [32], *A*. *dispersus* [33], four genera of Pentatomidae [36], and *S*. *furcifera* [34]. The CAM plays the role of mechanoreceptors without pore in their cuticular structures [74,76–80]. On *C*. *lividipennis* antennae of each development stage, CAM with a pore could be involved in gustatory function and be highly susceptible to humidity [53,81].

SP may find on the front leg taris, which is responsible for the perception of female signals that elicited the copulation behavior [82], as well as the ovipositor, which is involved in the oviposition process with stabbing mechanism [28]. The SP in *C*. *lividipennis* was similar morphology with those described on the antennae of *Triceratopyga calliphoroides* [29], which may be also involved in olfactory function [83–85].

We found no notable difference in the structure of antennae and antennal sensilla of *C*. *lividipennis* of each development stage, but a significant difference in the types and numbers of the antennal sensilla. The types of antennal sensilla in adult females and males were more than those in five different nymphal stages. The CHA III only occurred in males with a large number. We also observed differences between sexes in the numbers of MIC, BAS II, TRI II, TRI III and MPLA. Such differences may suggest to being related sex-specific differences in behavior, e.g., courtship and host recognition. The results could further the study of olfactory mechanisms involved in behavioral responses, including habitat searching, host location, host detection, host recognition host acceptance, oviposition, mating, and host discrimination. Future studies on the functional morphology of the antennal sensilla using TEM coupled with electrophysiological recordings will likely confirm the functions of different sensilla identified in this study.

## Supporting information

S1 File Informations in differentiating *Cyrtorhinus lividipennis* and *Tyttus chinensis*. (DOCX)
