# Supplementary material for: The structure and morphologic changes of antennae of Cyrtorhinus lividipennis (Hemiptera: Miridae: Orthotylinae) in different instars
Source: PLoS One. 2018 Nov 26;13(11):e0207551. doi: 10.1371/journal.pone.0207551 (PMC6261047; doi:10.1371/journal.pone.0207551)
Supplement: S1 File — (DOCX) [file pone.0207551.s001.docx]

Informations in differentiating *Cyrtorhinus lividipennis* and *Tyttus chinensis*:

*Cyrtorhinus lividipennis*: They have an elongated, green body, the membrane having two closed cells at the base. The hind wings are membranous and slightly shorter than the forewings. A pairs of strumae with green color were present in the anterior of pronotum; formed as two butterfly-shaped shoulder spots on posterior of the strumae; scutellum with a black brown longitudinal spot on the centre; embolium, cuneus, corium and clavus of hemelytra pale green.

*Tytthus chinensis*: They are yellowish when young and brown to dark brown when mature. Pronotum black or black brown, flat; scutellum black, flat, clothed with sparse hairs; hemelytra is tinge.


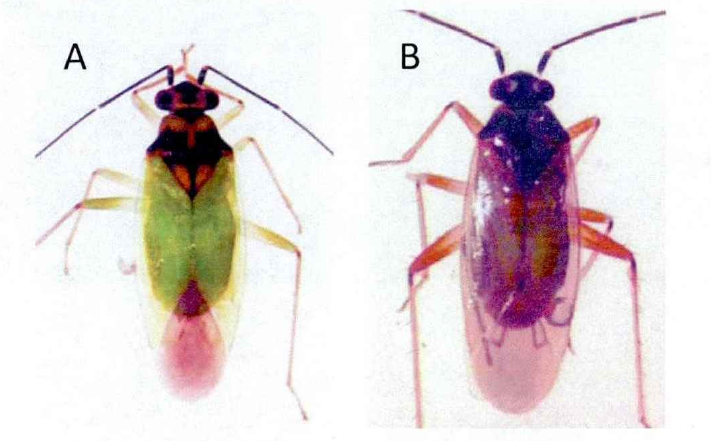


Figure 1 Adult *Cyrtorhinus lividipennis* (A) and adult *Tytthus chinensis* (B)


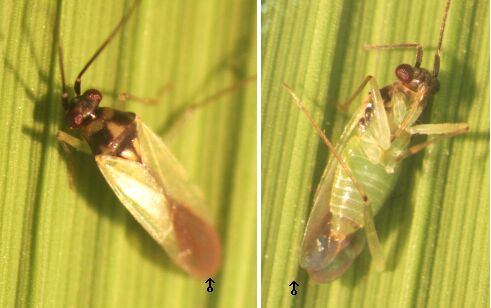


Figure 2 Adult male *Cyrtorhinus lividipennis* (A) Dorsal view; (B) ventral view


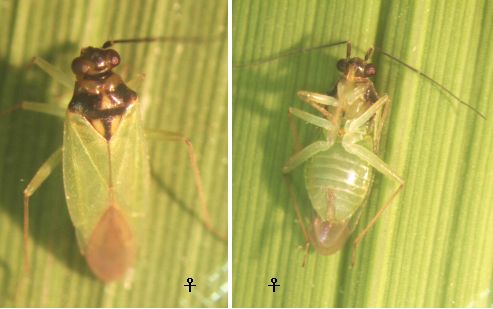


Figure 3 Adult female *Cyrtorhinus lividipennis* (A) Dorsal view; (B) ventral view
